# Solid-Phase Spectrometric Determination of Organic Thiols Using a Nanocomposite Based on Silver Triangular Nanoplates and Polyurethane Foam

**DOI:** 10.3390/s23187994

**Published:** 2023-09-20

**Authors:** Aleksei Furletov, Vladimir Apyari, Pavel Volkov, Irina Torocheshnikova, Stanislava Dmitrienko

**Affiliations:** 1 Department of Chemistry, Lomonosov Moscow State University, 119991 Moscow, Russia; 2 Scientific-Research Institute of Chemical Reagents and Special Purity Chemicals, National Research Center “Kurchatov Institute”, 107076 Moscow, Russia

**Keywords:** diffuse reflectance spectroscopy, nanocomposite materials, optical sensors, organic thiols, polyurethane foam, silver triangular nanoplates, visual colorimetry

## Abstract

Adsorption of silver nanoparticles on polymers may affect the processes in which they participate, adjusting the analytical characteristics of methods for the quantitation of various substances. In the present study, a composite material based on silver triangular nanoplates (AgTNPs) and polyurethane foam was proposed for chemical analysis. The prospects of its application for the solid-phase/colorimetric determination of organic thiols were substantiated. It was found that aggregation of AgTNPs upon the action of thiols is manifested by a decrease in the AgTNPs’ localized surface plasmon resonance band and its significant broadening. Spectral changes accompanying the process can be registered using household color-recording devices and even visually. Four thiols differing in their functional groups were tested. It was found that their limits of detection increase in the series cysteamine < 2-mercaptoethanol < cysteine = 3-mercaptopropionic acid and come to 50, 160, 500, and 500 nM, respectively. The applicability of the developed approach was demonstrated during the analysis of pharmaceuticals and food products.

## 1. Introduction

The option of preparing nanocomposite materials with specific optical properties opens the way to the creation of new solid-phase analytical reagents based on them. The use of such reagents is mainly associated with the production of an analytical signal in the solid phase. Therefore, it is most expedient to combine these reagents with solid-phase spectroscopy methods, in particular, diffuse reflectance spectroscopy and visual and digital colorimetry. The advantages of such nanocomposites are ease of use, ease of preparing solid-phase analytical tools, and ease of signal detection using cheap and affordable household color-recording devices, such as a monitor calibrator, a scanner, a digital camera, or a smartphone.

As a sensitive element of optical sensors, silver nanoparticles of different morphology are widely used [1,2,3,4,5,6,7,8]. It is known that aquasols of silver nanoparticles are intensely colored as a result of the localized surface plasmon resonance (LSPR) [9,10]. Researchers’ attention is mainly focused on the application of spherical silver nanoparticles in chemical analysis [11,12,13,14,15,16]. However, there is a remarkably smaller number of articles on the application of non-spherical silver nanoparticles, such as nanocubes [17,18], nanowires [19], nanodiscs [20], nanorods [21], and triangular nanoplates (AgTNPs) [22,23,24]. The higher sensitivity of AgTNPs with respect to a number of analytes could play a decisive role in the development of new optical sensors for chemical analysis. In recent decades, AgTNPs have become increasingly used for the determination of metal ions and anions, as well as organic and inorganic compounds [25]. It should be noted that the most promising direction seems to be the use of AgTNPs for the determination of compounds without chromophore groups, such as organic thiols. These compounds have no predisposition to be detected with classical colorimetric reagents. It should also be noted that most of the methods described for the determination of thiols require complex sample preparation, the use of expensive equipment, or high consumption of reagents. In this regard, it seems advisable to use various analytical systems based on silver nanoparticles, which have well proven themselves in the determination of many other analytes [26,27,28]. As a rule, anisotropic nanoparticles containing a large number of angles with uncompensated surface energy are more reactive, which led us to the choice of AgTNPs for further research. The importance of developing new procedures for the determination of organic thiols results from their significant role in biochemical processes in the human and animal organisms. Sulfur-containing functional groups are often found in the active sites of receptors, enzymes, and hormones. There is also evidence that organic thiols protect cell membranes and biologically active molecules from the destructive action of active oxygen radicals [29].

As carriers for the preparation of solid-phase analytical reagents, sorbents of various natures can be used, for example, paper [30], polymeric materials [31], silica gels [32], etc. In this study, we proposed polyurethane foam (PUF) as a matrix for obtaining new nanocomposite materials. The advantages of PUF include the high sorption capacity, chemical inertness, lightness, solidity, thermal stability, high availability, and low cost. The ability of PUF to sorb and firmly retain significant amounts of compounds of various classes was previously used in the development of methods for the sorption preconcentration of heavy metals and organic compounds from air and water and their determination after desorption or directly in the sorbent phase [33].

In this study, a new nanocomposite based on polyurethane foam and silver triangular nanoplates (AgTNPs/PUF) has been proposed for chemical analysis. The prospects of its application for the solid-phase detection of organic thiols have been evaluated.

## 2. Materials and Methods

### 2.1. Reagents and Instruments

To synthesize AgTNPs, the following reagents were used: silver(I) nitrate (PZTsM-Vtormet, Moscow, Russia, analytical grade), sodium citrate (Sigma-Aldrich, St. Louis, MO, USA, ≥99.5%), poly(1-ethenylpyrrolidin-2-one) (Acros Organics, Waltham, MA, USA, M.W. 58,000 g mol^−1^, 99%), hydrogen peroxide (Sigma-Aldrich, St. Louis, MO, USA, 30 wt.% in H_2_O, ACS), and sodium borohydride (Acros Organics, Waltham, MA, USA, 99%).

Aggregation of AgTNPs on PUF in the presence of organic thiols was studied using cysteamine hydrochloride (Sigma-Aldrich, St. Louis, MO, USA, ≥97.0%), *L*-cysteine (Aldrich, St. Louis, MO, USA, >97%), 3-mercaptopropionic acid (Aldrich, St. Louis, MO, USA, ≥99%), and 2-mercaptoethanol (Aldrich, St. Louis, MO, USA, ≥99.0%).

Besides, *L*-alanine (Sigma-Aldrich, St. Louis, MO, USA, ≥99.0%), *DL*-valine (Sigma-Aldrich, St. Louis, MO, USA, ≥97%), *L*-isoleucine (Sigma-Aldrich, St. Louis, MO, USA, ≥99.5%), *DL*-serine (Sigma-Aldrich, St. Louis, MO, USA, ≥98%), *L*-tyrosine (Sigma-Aldrich, St. Louis, MO, USA, ≥99.0%), *L*-phenylalanine (Sigma-Aldrich, St. Louis, MO, USA, ≥99.0%), nitric acid (OJSC NAC Azot, Novomoskovsk, Russia, 68% aqueous solution, chemically pure grade), acetone (Sigma-Aldrich, St. Louis, MO, USA, ≥99.5%), acetonitrile (Sigma-Aldrich, St. Louis, MO, USA, ≥99.9%, HPLC grade), sodium hydroxide (Reakhim LLC, Moscow, Russia, chemically pure grade), sodium nitrate (Reakhim LLC, Moscow, Russia, chemically pure grade), hydrochloric acid (Reakhim LLC, Moscow, Russia, 36% aqueous solution, chemically pure grade), and glacial acetic acid (IREA 2000 LLC, Moscow, Russia, chemically pure grade) were also used in the present study.

Polyurethane foam based on ethers (Dagmar, Saint Petersburg, Russia) was used as a matrix for the preparation of solid-phase analytical reagents based on AgTNPs. The sorbent was used in the form of tablets 5 mm height, 16 mm in diameter, and weighing (20 ± 2) mg. To remove impurities, PUF tablets were washed twice in acetone (each wash for 10 min), after which they were dried to an air-dry state.

Substances were weighed on an Adventurer analytical balance (OHAUS, Parsippany, NJ, USA). Aliquots of liquid substances were collected using automatic single-channel variable-volume pipette Discovery Comfort dispensers (HTL, Warsaw, Poland). Solutions were mixed in polypropylene test tubes manufactured by ISOLAB (Eschau, Germany). Solutions were shaken using Ekros PE-6500 electromechanical vibratory mixer (Ekros, Russia). Diffuse reflectance spectra in the visible region of the spectrum were recorded using an i1 Pro 2 mini-spectrophotometer (X-Rite, Grand Rapids, MI, USA). To measure pH, an Expert-001 pH meter-ionomer (Ekoniks-Expert, Moscow, Russia) was used.

Chromatographic determination of cysteine in samples was carried out using a Tsvet Yauza high performance liquid chromatograph (Khimavtomatika, Voronezh, Russia) with an amperometric detector (*E* = 1.2 V). Silica with chemically grafted octadecyl groups was used as the stationary phase. It was packed in a Luna 5u C18 chromatographic column (Phenomenex, Torrance, CA, USA) equipped with a Security Guard C18 guard column (Phenomenex, USA). As the mobile phase, a mixture consisting of 50 vol. % acetonitrile and 50 vol. % deionized water was used. The volume of sample injected into the liquid chromatograph was equal to 20 μL. The sample was injected using a dosing loop. The flow rate of the mobile phase was equal to 0.40 mL min^–1^. To prepare the eluent, acetonitrile was filtered through a Ftoroplast 0.2 µm membrane filter (BioKhimMak ST, Moscow, Russia) using a Millipore high-performance vacuum pump (Merck, Germany). The eluents were degassed in a Bransonic 1510R-DTH ultrasonic bath (Branson Ultrasonics Corporation, Brookfield, CT, USA).

Electron microscopic studies of silver triangular nanoplates on the surface of polyurethane foam were carried out using a JSM 7100 F scanning electron microscope (Jeol, Tokyo, Japan). The accelerating voltage of the electron gun was chosen in the range of 10–20 kV. The SEM images were obtained in secondary electrons and stored in a digitized form.

### 2.2. Preparation of Label-Free AgTNPs

Preparation of label-free silver triangular nanoplates was carried out as previously described by G.S. Métraux and C.A. Mirkin [34]. Briefly, 0.5 mL of 10 mM silver(I) nitrate solution was diluted with 4.3 mL of deionized water. Then, 2.3 mL of 1% sodium citrate solution, 0.6 mL of 20 g L^−1^ poly(1-ethenylpyrrolidin-2-one) solution, and 1.2 mL of 3% hydrogen peroxide solution were successively added into the reaction mixture under vigorous stirring. Then, 1.0 mL of freshly prepared 35 mM NaBH_4_ solution was dropwise added to the reaction mixture. It became a pale yellow-green color, which 30 min later changed to blue-violet. After that, the stirring was stopped. The as-prepared AgTNPs aquasol was stored at room temperature. Additional purification of the synthesized nanoparticles before loading to polyurethane foam was not carried out. A TEM image of AgTNPs is provided in the Supplementary Materials (Figure S1).

### 2.3. Preparation of AgTNPs/PUF Composite Material

To prepare the composite material based on AgTNPs and polyurethane foam, PUF tablets were placed into 10 mL of an aqueous solution containing 0.16 mM AgTNPs, squeezed with a glass rod, and shaken on an electromechanical shaker for 30 min. After that, the colored tablets were removed and dried between sheets of filter paper.

Determination of AgTNPs content in the initial and equilibrium solutions was carried out with UV–Vis spectrometry according to a pre-built calibration graph. The amount of adsorbed nanoparticles (*a*, μmol Ag g^–1^) was assessed from the decrease in the concentration of AgTNPs in aqueous solution according to the formula

$$a=\frac{\left(c_{0}-\left[c\right]\right)\cdot V}{m}$$

where *c*_0_ is the initial concentration of AgTNPs, mM Ag; [*c*] is the equilibrium concentration of AgTNPs, mM Ag; *V* is the volume of the solution from which sorption was carried out, mL; *m* is the exact mass of the PUF tablet, g.

The content of silver nanoparticles in the polyurethane phase was evaluated by the value of the Gurevich–Kubelka–Munk function *F*(*R*) at a wavelength corresponding to the maximum of AgTNPs LSPR band:

$$F\left(R\right)=\frac{{\left(1-R\right)}^{2}}{2\cdot R}$$

where *R* is the diffuse reflection coefficient.

To get a value directly proportional to the specific adsorption, we calculated the difference

$$∆F\left(R\right)=F\left(R\right)-F_{0}\left(R\right)$$

where *F*(*R*) is the value of the Gurevich–Kubelka–Munk function for the PUF tablet modified with silver nanoparticles, *F*_0_(*R*) is the value of the Gurevich–Kubelka–Munk function for unmodified PUF tablet.

### 2.4. Diffuse Reflection Measurement

Diffuse reflection of AgTNPs/PUF in the visible spectral region was recorded using an i1 Pro 2 mini-spectrophotometer. This device was previously proposed by our research group as a convenient alternative to a diffuse reflectance spectrometer [35]. It is a miniature USB-compatible device with a holographic grating as a monochromator and a 128-pixel LED array as a detector. It has a built-in gas-filled tungsten lamp as a D50 type radiation source. The i1 Pro 2 mini-spectrophotometer allows the measuring of diffuse reflection, *R*, in the wavelength range from 380 to 730 nm with 10 nm increments, as well as colorimetric characteristics of samples, for example, color coordinates in RGB, CMYK, or Lab systems.

To carry out measurements, the i1 Pro 2 mini-spectrophotometer was connected to the USB port of a personal computer running the Microsoft Windows 10 operating system, and the device was calibrated on a white substrate included in the standard package. After that, the colored samples were measured, and the diffuse reflection coefficients for various wavelengths were exported to the Microsoft Excel 2019 program.

### 2.5. Procedures

To plot integral kinetic curves for the interaction between the nanocomposite and organic thiols, a certain amount of analyte was introduced into polypropylene test tubes and the acetate buffer solution was added up to a volume of 5.00 mL. Then, the AgTNPs/PUF tablets were placed into the test tubes, squeezed with a glass rod, and shaken for a specified time. After the specific time, AgTNPs/PUF tablets were removed, dried between sheets of filter paper, and the diffuse reflectance spectra of nanocomposites were recorded. In all cases, the value of the Gurevich–Kubelka–Munk function change at the maximum of AgTNPs LSPR band (Δ*F*) was used as an analytical signal.

To study the effect of pH, the selected organic thiol was introduced into the test tubes and the final volume of the solution was adjusted to 5.00 mL with solutions of 0.1 M CH_3_COOH and 0.1 M NaOH in various ratios. Then, we proceeded as described above.

To plot calibration curves, different amounts of analytes were introduced into test tubes and the final volume of the solution was adjusted to 5.00 mL with the acetate buffer solution. Then, we proceeded as described above.

### 2.6. Sample Pretreatment

The “Perfalgan” pharmaceutical for infusions (Bristol Myers Squibb PJSC, Anagni, Italy) was diluted 100 times with deionized water. For the quantitation, an aliquot portion of this solution was taken. One tablet of the biologically active food additive “NAC Complex” (Nittany Pharmaceuticals Inc., Milroy, PA, USA) or one tablet of brewer’s yeast (Ekko Plus LLC, Moscow, Russia) were dissolved in 30 mL of deionized water using sonication for 15 min. The solution was filtered through a pleated paper filter into a 50 mL flask and brought to the mark with deionized water. An aliquot part of this solution was appropriately diluted and taken for analysis.

## 3. Results and Discussion

### 3.1. Characteristics of AgTNPs/PUF

**Figure 1:**
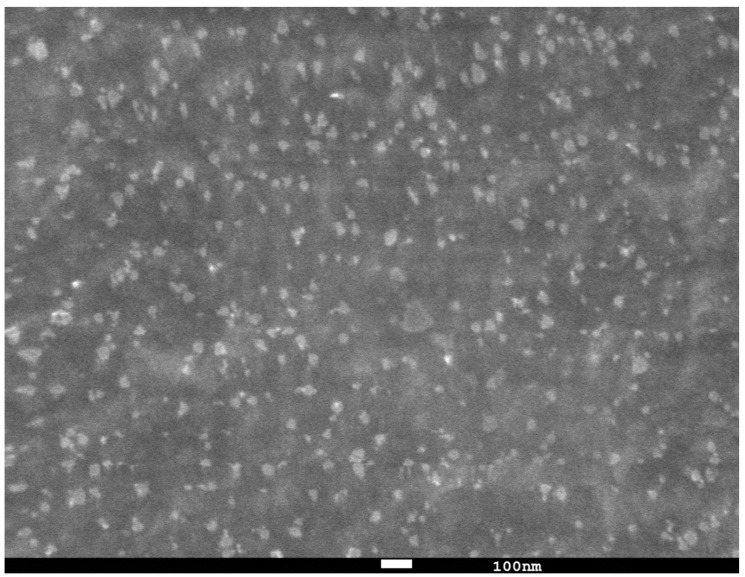
SEM image of silver triangular nanoplates on the surface of polyurethane foam (magnification 50,000 times).

The nanocomposite obtained in the present study was characterized using scanning electron microscopy (SEM) (Figure 1). It indicates that AgTNPs are located on the surface of polymer membranes. The degree of their aggregation on the polyurethane foam is rather low and the nanoparticles are predominantly separated from each other. Electron diffraction from an individual silver triangular nanoplate is presented in the Supplementary Materials (Figure S2).

**Figure 2:**
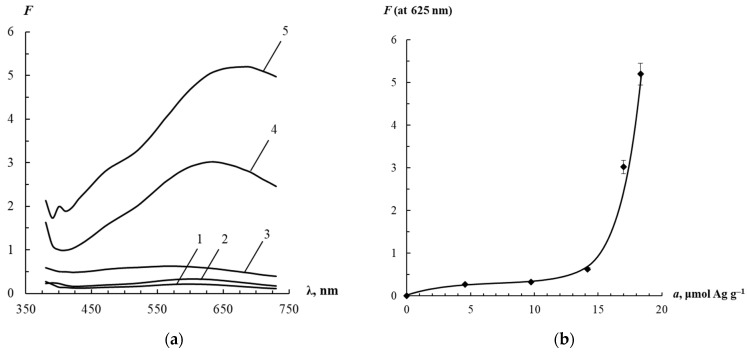
(**a**) Diffuse reflectance spectra of silver triangular nanoplates on the surface of polyurethane foam. Specific adsorption values *a*, µmol Ag g^–1^: 4.6 (1), 9.7 (2), 14.2 (3), 17.0 (4), 18.3 (5); (**b**) Dependence of the change in the Gurevich–Kubelka–Munk function at 625 nm on the specific adsorption.

It should be mentioned that the composite material based on AgTNPs and PUF has a blue color due to the LSPR phenomenon. The diffuse reflection spectra of the AgTNPs/PUF composite are shown in Figure 2a. As can be seen, nanoparticles on the surface of PUF retain the ability to have an LSPR band, so these nanocomposite materials may be of interest for use in chemical analysis as optical sensors.

It was found that, as the concentration of AgTNPs on the PUF surface increases, the maximum of the LSPR band shifts to the long-wavelength region, and its amplitude gradually increases. To elucidate the reason for the described spectral changes, we studied the dependence of the Gurevich–Kubelka–Munk function at 625 nm on the specific adsorption of AgTNPs (Figure 2b). This dependence is characterized by the presence of two linear sections in the regions of low and high values of specific adsorption. This fact indicates a sharp change in the molar absorption coefficient of AgTNPs on PUF surface near 15 μmol Ag g^–1^. At a specific adsorption of about 15 μmol Ag g^–1^, the curve has a fracture, which is presumably associated with the onset of self-organization of individual nanoparticles into stacks (Figure S1b in the Supplementary Materials). This circumstance probably leads to a change in the mechanism of AgTNPs sorption on PUF and explains the shift of the LSPR band in the diffuse reflection spectra to the long wavelength region.

### 3.2. Interaction between AgTNPs/PUF and Organic Thiols

It is known from the literature data that the processes of aggregation of silver nanoparticles in solution can be used to determine organic thiols [36]. The immobilization of silver nanoparticles on the surface of polyurethane foam should affect the aggregation processes with their participation. In its turn, it may affect the sensitivity and selectivity of the determination of organic thiols. Therefore, it was of interest to study the possibility of using AgTNPs/PUF nanocomposite as a solid-phase reagent for the determination of organic thiols as well as to reveal the behavior of AgTNPs in the PUF matrix compared to aquasols of AgTNPs.

**Figure 3:**
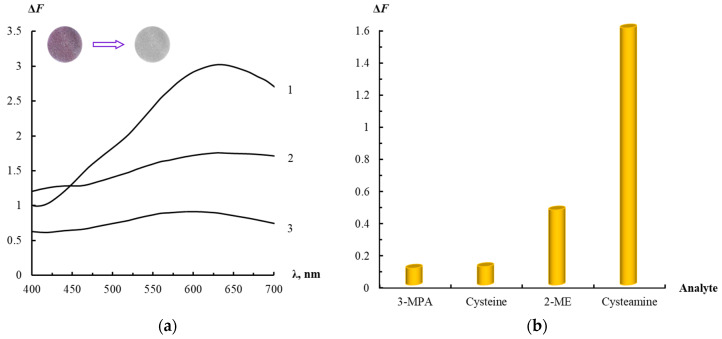
(**a**) Diffuse reflectance spectra of AgTNPs/PUF composite at different concentrations of cysteine. *c*(cysteine), μM = 0 (1), 17 (2), 35 (3), *c*(AgTNPs) = 17 μmol Ag g^–1^, pH 5.0, *t* = 40 min. Inset: change in the color of the nanocomposite upon interaction with thiols; (**b**) Change in the diffuse reflectance of AgTNPs/PUF composite depending on the nature of the organic thiol. *c*(AgTNPs) = 17 μmol Ag g^–1^, *c*(thiol) = 0.2 mg L^–1^, pH 5.0, *t* = 40 min.

To achieve this goal, four organic thiols have been studied (Table 1). These thiols have a similar chemical structure, but different functionality due to the presence of additional functional groups. The different functionality was necessary to reveal the relationship between the structural features of the organic thiol and its ability to interact with the nanocomposite. Diffuse reflection spectra of the AgTNPs/PUF composite after interaction with the selected organic thiols were recorded in accordance with the procedure described in Section 2.4 and Section 2.5. The spectra for cysteine are presented in Figure 3a. The spectra are similar for cysteamine, 3-mercaptopropionic acid, and 2-mercaptoethanol.

The intensity of the LSPR band of AgTNPs on the polyurethane surface decreases with the increase in the thiol concentration. In addition, its broadening was also observed, which was presumably associated with AgTNP aggregation. These spectral changes were accompanied by a change in the color of the nanocomposite, which opens up the possibility of detecting organic thiols visually.

Some important features of AgTNP aggregation on PUF induced by organic thiols were evaluated. It was found that the structure of the analyte and the nature of additional functional groups have a direct effect on the analytical signal (Figure 3b). The greatest spectral changes are induced by the positively charged organic thiol, namely, cysteamine. For the electrically uncharged 2-mercaptoethanol, the signal is lower. When interacting with cysteine and 3-mercaptopropionic acid, the spectral characteristics of the nanocomposite change insignificantly, which may result from the presence of a negatively charged functional group at pH 5.0 in the structure of the analytes. Thus, we can suppose that the aggregation of AgTNPs is promoted with an increase in the number of positively charged functional groups (for example, –NH_3_^+^) and a decrease in the number of negatively charged functional groups (for example, –COO^–^). This can be achieved not only by varying the nature of the analyte, but also by changing the reaction conditions, mainly the pH of the medium.

**Figure 4:**
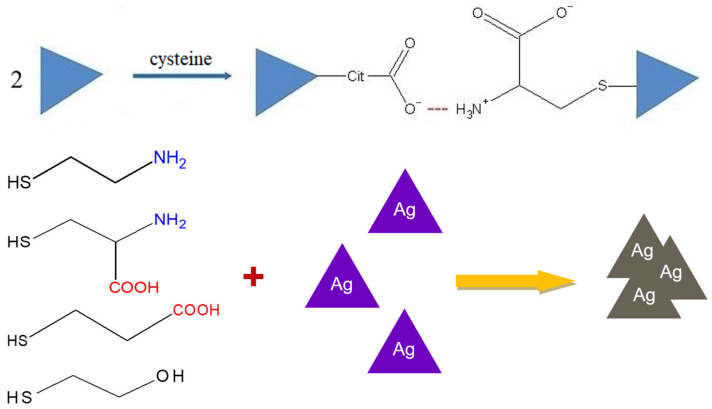
Probable mechanism of silver triangular nanoplate aggregation under the influence of thiols (in the example of cysteine, Cit is the citrate-ion residue).

The mechanism of AgTNP aggregation under the influence of cysteine has been proposed by Y. Li and co-authors [37]. According to this paper, the mechanism includes the formation of cysteine–cysteine intermolecular hydrogen bonds between carboxyls and protonated amino groups of two opposite molecules. However, the fact that similar aggregative phenomena are observed in the case of thiols without carboxyl groups (cysteamine), amino groups (3-mercaptopropionic acid) or both (2-mercaptoethanol) allows us to assume that the interaction mechanism probably involves the participation of citrate ions on the surface of AgTNPs in the intermolecular bond formation (Figure 4).

To optimize the conditions for the determination of analytes, we have studied the effect of the interaction time and solution pH on the decrease in the intensity of the LSPR band of nanoparticles.

#### 3.2.1. Effect of Interaction Time

**Figure 5:**
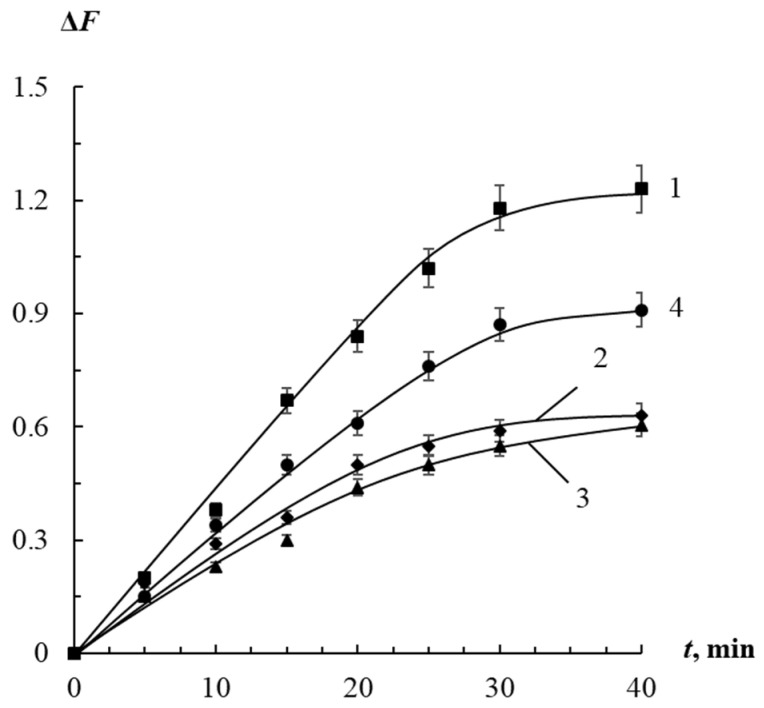
Change in the diffuse reflectance of AgTNPs/PUF composite in a solution containing cysteamine (1), cysteine (2), 3-mercaptopropionic acid (3), and 2-mercaptoethanol (4), depending on the interaction time. *c*(AgTNPs) = 17 μmol Ag g^–1^, pH 5.0. (1) *c*(cysteamine) = 2 μM; (2) *c*(cysteine) = 10 μM; (3) *c*(3-MPA) = 10 µM; (4) *c*(2-ME) = 5 μM.

The influence of the interaction time on the sensitivity of the determination of organic thiols was studied. It was found that the maximum value of the analytical signal is reached in 40 min after the addition of organic thiols to AgTNPs/PUF composite (Figure 5). A SEM image of the surface of the nanocomposite material after its interaction with organic thiols is shown in Figure S3 in the Supplementary Materials. In the SEM image, there are practically no silver nanoparticles on PUF, which confirms that 40 min is sufficient to complete the interaction.

In general, the reaction proceeds much more slowly on the surface of the solid phase than in a colloidal solution, where this process takes from 2.5 to 15 min [36]. In addition, the kinetic curves lack a segment in the region of short times corresponding to the rapid stage of small aggregates formation. Both of these effects are a consequence of the immobilization of AgTNPs on the surface of PUF, which limits their mobility and reduces reactivity.

#### 3.2.2. Effect of pH

**Figure 6:**
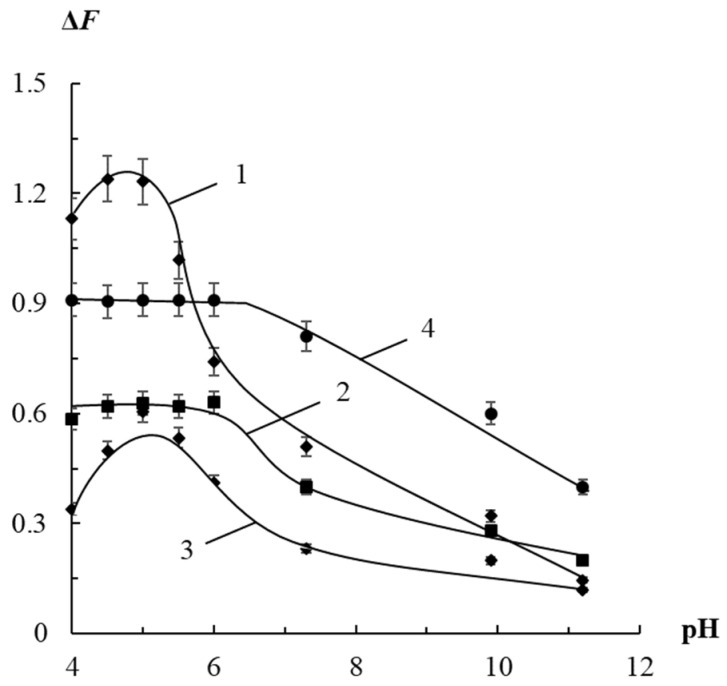
Change in the diffuse reflectance of AgTNPs/PUF composite in a solution containing cysteamine (1), cysteine (2), 3-mercaptopropionic acid (3) and 2-mercaptoethanol (4), depending on the pH value. *c*(AgTNPs) = 17 μmol Ag g^–1^, *t* = 40 min. (1) *c*(cysteamine) = 2 μM; (2) *c*(cysteine) = 10 μM; (3) *c*(3-MPA) = 10 µM; (4) *c*(2-ME) = 5 μM.

The influence of pH on the sensitivity of the quantitation of organic thiols was studied. It was found that the maximum value of the analytical signal is achieved in the pH range of 4.5–5.0 for cysteamine, and in the range of 4.5–6.0 for cysteine, 3-mercaptopropionic acid, and 2-mercaptoethanol (Figure 6). It should be noted that these results are quite similar to the behavior of AgTNPs in a colloidal solution.

These observations indicate that protonation of citrate ions on the AgTNPs surface contributes to their aggregation under the influence of organic thiols, which most strongly affects the interaction with compounds of the cationic type. The interaction with thioacids as well as non-ionizing analytes is also possible under the conditions of dissociation of carboxyl groups.

### 3.3. Determination of Organic Thiols Using Solid-Phase Spectrometry

#### 3.3.1. Analytical Performance

Under the selected experimental conditions, some analytical features of the developed techniques for quantitation of organic thiols using AgTNPs/PUF have been assessed (Table 2). Limits of detection (LODs) were evaluated using 3σ criteria, and limits of quantitation (LOQs) were assessed using 10σ criteria. It was found that limits of detection increase in the series cysteamine < 2-mercaptoethanol < cysteine = 3-mercaptopropionic acid and come to 50, 160, 500, and 500 nM, respectively. The lower limit of the determination range was taken to be equal to the LOQ value, and the upper limit of the determination range was estimated as the maximum analyte concentration at which the calibration curve remained linear. It was found that the ranges of linearity of the calibration graphs are about an order of magnitude. The relative standard deviation (RSD) corresponding to the middle of the determination range does not exceed 5%.

#### 3.3.2. Interference Studies

To evaluate the possible interfering effect of foreign compounds on the determination of organic thiols, aqueous solutions with different ratios of organic thiol and foreign compounds were prepared. It was found that the detection of analytes was not affected by 4000-fold quantities of Na^+^, K^+^, Mg^2+^, Ca^2+^, Sr^2+^, Ba^2+^, CH_3_COO^−^, 2000-fold quantities of Al^3+^ and NO_3_^−^, and 1000-fold quantities of Cr^3+^, Co^2+^, Cl^−^, and SO_4_^2−^. It is worth noting that 3000-fold quantities of some proteinogenic amino acids (namely, *L*-alanine, *DL*-valine, *L*-isoleucine, *DL*-serine, *L*-tyrosine, and *L*-phenylalanine) did not interfere with the determination of organic thiols. This is understandable if we consider the interaction mechanism based on the formation of a stable bond between the thiol group of the analyte and silver atoms of AgTNPs. However, the approach proposed in this study does not have intragroup selectivity and does not allow the determination of some thiols over others.

#### 3.3.3. Sample Analysis

The assessment of the applicability of the developed approach was carried out using real samples containing cysteine as the main component. The choice of cysteine was due to its relatively wide distribution and significant biological role. The “Perfalgan” pharmaceutical for infusions (Bristol Myers Squibb PJSC, Italy), biologically active food additive “NAC Complex” (Nittany Pharmaceuticals Inc., Milroy, PA, USA), and brewer’s yeast (Ekko Plus LLC, Russia) were used as the samples for analysis. The results of the determination are presented in Table 3. To confirm the accurate determination of cysteine, all samples were further analyzed using HPLC. The content of the analyte found using the proposed approach, considering the confidence interval, coincides with the content found using the reference method. This fact testifies to the good accuracy of the determination results.

#### 3.3.4. Comparison with Existing Methods

Analytical features of the proposed and existing methods for the quantitation of cysteamine and cysteine, as the most often determined thiols, are presented in Table 4. From a comparison of the presented features, it can be concluded that the developed technique has a fairly good sensitivity. The detection limits of cysteamine and cysteine are either comparable or better than similar values for the vast majority of the existing methods. The limitation of the proposed approach is the relatively low selectivity, inferior to such methods as HPLC or spectrofluorimetry. However, at the same time, it does not require the use of expensive equipment, which is its important advantage.

It can be supposed that the proposed approach can be used, for example, to carry out non-laboratory analysis of some pharmaceuticals or food products. This is possible thanks to the convenient form of the solid analytical reagent, which can be carried anywhere. Another assumed area for using the developed approach is “in-house analysis”. To register a colorimetric analytical signal, one can use household color-recording devices that are widely accessible, for example, a smartphone, a digital scanner, or camera, as well as a monitor calibrator. Another area of potential application of the developed approach is test methods of analysis. With the increase in the number of chemical analyses carried out, screening methods become of great importance, allowing a relatively simple and quick preliminary assessment of the content of target substances to decide on the advisability of the further detailed analysis using powerful methods of analytical chemistry. Due to the advantages described above, the nanocomposite material based on AgTNPs and PUF can be successfully used for preliminary screening of samples.

## 4. Conclusions

In this study, AgTNPs/PUF nanocomposite material has been proposed for chemical analysis. This material combines the high sensitivity of AgTNPs and the good sorption and performance properties of PUF. It has been revealed that AgTNPs in a PUF matrix retain the capability for localized surface plasmon resonance, which can be used to develop new colorimetric reagents for solid-phase spectroscopy.

The prospects of AgTNPs/PUF application for the solid-phase/colorimetric determination of organic thiols have been substantiated. Changes in the color of nanocomposites observed with the naked eye make it possible to use this material for the visual colorimetric determination of thiols. The proposed interaction mechanism consist in the aggregation of AgTNPs due to the electrostatic interaction of citrate ions located on the surface of silver nanoparticles with the functional groups of the studied thiols.

Compared to a colloidal solution of AgTNPs, the nanocomposite material is inferior in sensitivity to organic thiols. Despite this, AgTNPs/PUF still exhibits high sensitivity to thiols compared to many of the methods described in the literature. The developed approach is also characterized by high selectivity in the presence of inorganic ions and proteinogenic amino acids, which makes it possible to improve the selectivity of thiol determination compared to the usage of AgTNP colloidal solution. Other advantages of the nanocomposite material are ease of use and possibility of using a cheap and compact mini-spectrophotometer to record an analytical signal.

## Figures and Tables

**Table 1:** Organic thiols studied in this work.

Chemical Name	MW, g mol^–1^	Structure	Form of Existence of the Compound at pH 5 *
Cysteamine	77.15	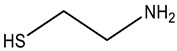	Cation
Cysteine	121.16	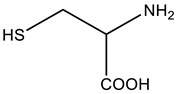	Zwitterion
3-Mercaptopropionic acid (3-MPA)	106.14	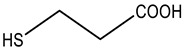	Anion
2-Mercaptoethanol (2-ME)	78.13	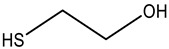	Uncharged molecule

* Form of existence of the compound was predicted using ACD Labs 6.00 software (Canada).

**Table 2:** Analytical features of the determination of organic thiols.

Analyte	Calibration Curve Equation (*c*, μM)	LOD, μM	LOQ, μM	Determination Range, μM	RSD *, %	RSD **, %
Cysteamine	Δ*F* = 0.616 × *c* (*r*^2^ = 0.987)	0.05	0.13	0.13–3	5	13
2-Mercaptoethanol	Δ*F* = 0.182 × *c* (*r*^2^ = 0.989)	0.16	0.5	0.5–10	4	11
Cysteine	Δ*F* = 0.063 × *c* (*r*^2^ = 0.990)	0.5	1.5	1.5–35	4	11
3-Mercaptopropionic acid	Δ*F* = 0.061 × *c* (*r*^2^ = 0.991)	0.5	1.5	1.5–35	4	11

* Calculated for the middle of the determination range. ** Calculated for the limit of quantitation value.

**Table 3:** Results of cysteine detection in pharmaceuticals and food products (*n* = 3, *p* = 0.95).

Sample	Present Method		Reference Method *	
	Found	RSD, %	Found	RSD, %
“Perfalgan”	(0.15 ± 0.03) mg mL^–1^	8	(0.16 ± 0.02) mg mL^–1^	5
“NAC Complex”	(219 ± 40) mg per tablet	7	(211 ± 9) mg per tablet	2
Brewer’s yeast	(44 ± 8) mg per tablet	7	(50 ± 7) mg per tablet	6

* Reversed-phase HPLC with amperometric detection.

**Table 4:** Analytical features of the proposed and existing methods for the quantitation of cysteamine and cysteine.

Analyte	Method	LOD, μM	Determination Range, μM	Reference
Cysteamine	Voltammetry	4	10–100	[38]
	Spectrofluorimetry	0.35	2–16	[39]
	Voltammetry	0.09	0.3–450	[40]
	Spectrofluorimetry	0.07	2–10	[41]
	UV–Vis spectrometry	0.03	0.13–1.3	[36]
	UV–Vis spectrometry	0.005	0.10–1.0	[14]
	Diffuse reflectance spectrometry	0.05	0.13–3	This study
Cysteine	HPLC-UV	—	0.02–0.3	[42]
	Spectrofluorimetry	6.5	6.5–400	[43]
	UV–Vis spectrometry	3	3–100	[44]
	Voltammetry	0.6	—	[45]
	UV–Vis spectrometry	0.4	0.5–4	[46]
	UV–Vis spectrometry	0.05	0.17–2	[36]
	Diffuse reflectance spectrometry	0.5	1.5–35	This study

## Data Availability

The data presented in this study are available on request from the corresponding author.

## Supplementary Materials

The following supporting information can be downloaded at: https://www.mdpi.com/article/10.3390/s23187994/s1, Figure S1: (a) TEM image of individual silver triangular nanoplates; (b) TEM image of stacks consisting of silver triangular nanoplates; Figure S2: Electron diffraction from an individual silver triangular nanoplate; Figure S3: SEM image of the surface of nanocomposite material after its interaction with organic thiols (magnification 50,000 times).
