# Supplementary material for: Solid-Phase Spectrometric Determination of Organic Thiols Using a Nanocomposite Based on Silver Triangular Nanoplates and Polyurethane Foam
Source: Sensors (Basel). 2023 Sep 20;23(18):7994. doi: 10.3390/s23187994 (PMC10536471; doi:10.3390/s23187994)
Supplement: Supplementary file 1 [file sensors-23-07994-s001.zip › sensors-2593415-supplementary.pdf]

Supplementary Materials

Solid-Phase Spectrometric Determination of Organic Thiols Using a Nanocomposite Based on Silver Triangular Nanoplates and Polyurethane Foam

Aleksei Furletov, Vladimir Apyari, Pavel Volkov, Irina Torocheshnikova and Stanislava Dmitrienko

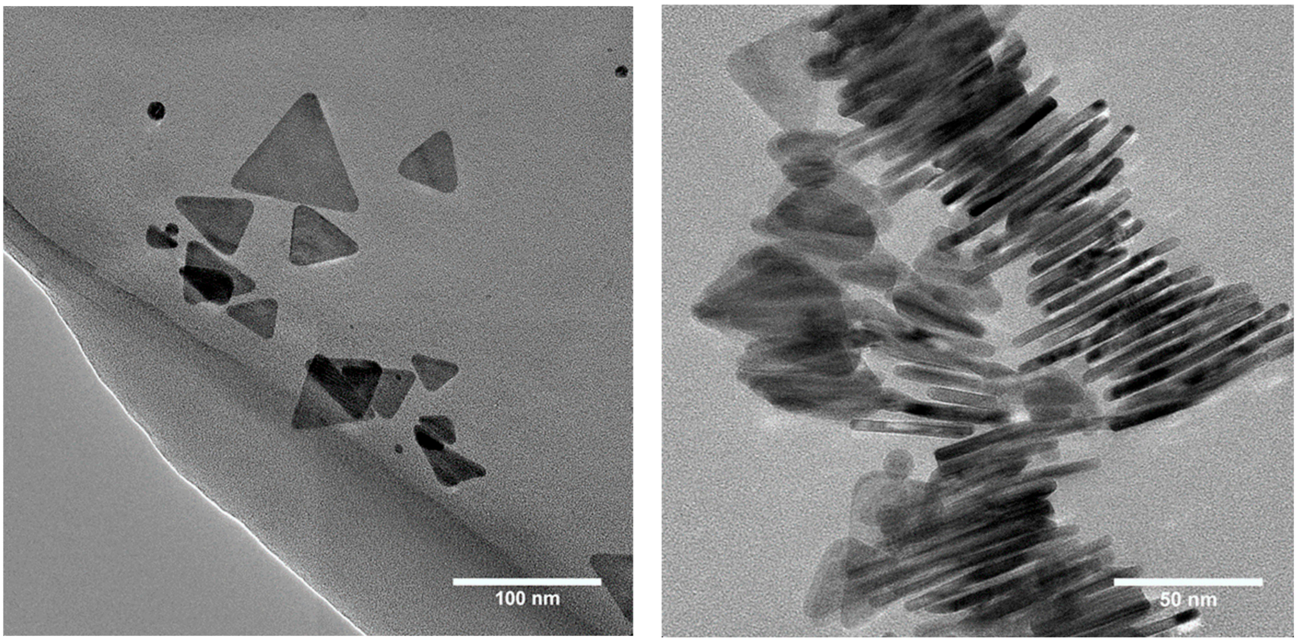

(**a**) (**b**)

Figure S1. (**a**) TEM image of individual silver triangular nanoplates; (**b**) TEM image of stacks consisting of silver triangular nanoplates.

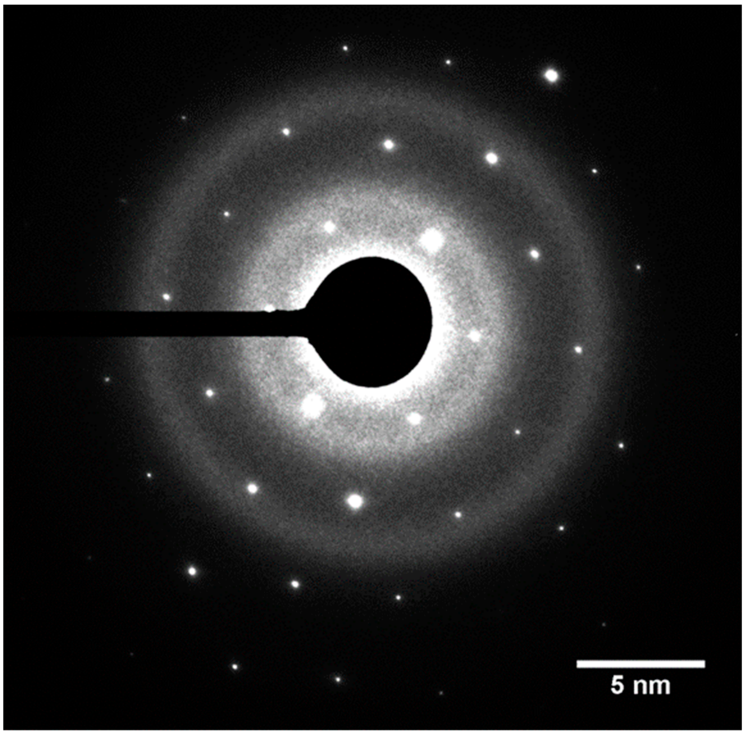

Figure S2. Electron diffraction from an individual silver triangular nanoplate.

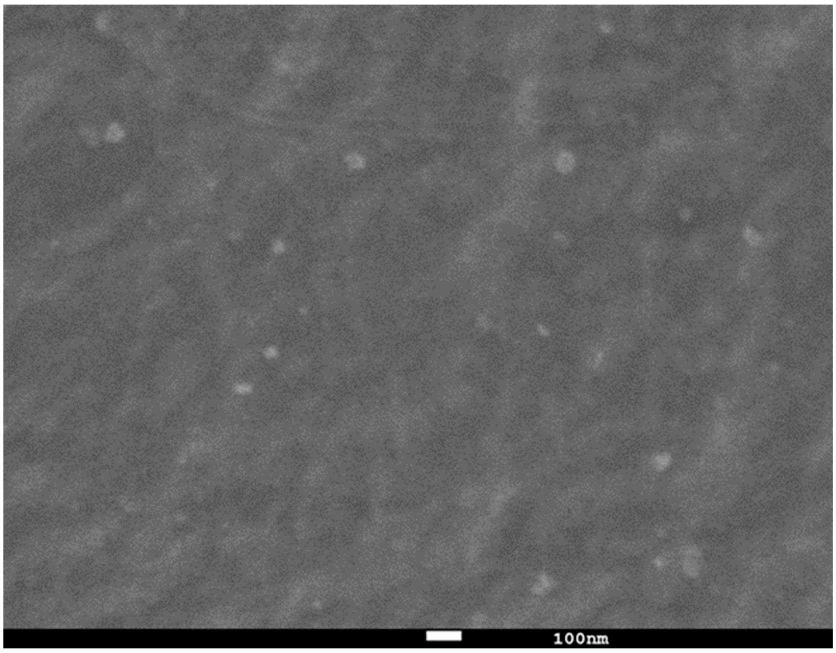

Figure S3. SEM image of the surface of nanocomposite material after its interaction with organic thiols (magnification 50,000 times).
